# The effect of a behavioral activation program on improving mental and physical health complaints associated with radiation stress among mothers in Fukushima: a randomized controlled trial

**DOI:** 10.1186/s12889-016-3819-x

**Published:** 2016-11-08

**Authors:** Kotaro Imamura, Yuki Sekiya, Yumi Asai, Maki Umeda, Naoko Horikoshi, Seiji Yasumura, Hirooki Yabe, Tsuyoshi Akiyama, Norito Kawakami

**Affiliations:** 1 Department of Mental Health, Graduate School of Medicine, The University of Tokyo, 7-3-1, Hongo, Bunkyo-ku, Tokyo, 113-0033 Japan; 2 Graduate School of Public Health Nursing, St Luke’s International University, Tokyo, Japan; 3 Radiation Medical Science Center for the Fukushima Health Management Survey, Fukushima Medical University School of Medicine, Fukushima, Japan; 4 Department of Public Health, Fukushima Medical University School of Medicine, Fukushima, Japan; 5 Department of Neuropsychiatry, Fukushima Medical University School of Medicine, Fukushima, Japan; 6 Department of Neuropsychiatry and Department of Psychosomatic Medicine, Kanto Medical Center NTT EC, Tokyo, Japan

**Keywords:** Behavioral activation, Psychological distress, Physical symptoms, Radiation anxiety, Psychological well-being

## Abstract

**Background:**

Mothers living with small children in Fukushima prefecture may experience radiation anxiety and related symptoms after the Fukushima Dai’ich Nuclear Power Plant Accident. A behavioral activation (BA) program was developed to improve their psychosomatic symptoms. The purpose of this randomized controlled trial was to examine the effectiveness of a BA program for improving psychological distress and physical symptoms among mothers with preschool children in Fukushima-prefecture 3 years after the Fukushima Daiichi Nuclear Power Plant accident.

**Methods:**

Participants were recruited from mothers living with a preschool child(ren) in Fukushima city and surrounding areas though a newspaper advertisement, posters, and flyers. Participants allocated to the intervention group received a newly developed group-based BA program, which consisted of two 90- min lessons with a 1-week interval. Psychological distress and physical symptoms as a primary outcome, and radiation anxiety and positive well-being (liveliness and life satisfaction) as a secondary outcome, were measured at baseline, 1- and 3-month follow-ups.

**Results:**

Participants were randomly allocated to either an intervention or a control group (18 and 19, respectively). The BA program showed a marginally significant intervention effect on psychological distress (*p* = 0.051) and physical symptoms (*p* = 0.07) at 1-month follow-up, while the effect became smaller at 3-month follow-up. The effect sizes at 1-month were medium to large (-0.72 and -0.56, respectively). There was a significant intervention effect on increasing liveliness at 3-month follow-up (*p* = 0.02); and there were marginally significant effects on life satisfaction at 1- and 3-month follow-ups (both *p* = 0.09).

**Conclusions:**

This BA program may be effective for improving psychological distress, physical symptoms, and well-being, at least for a short duration, among mothers with preschool children after the nuclear power plant accident in Fukushima, while a further large-scale study is needed.

**Trial registration:**

The UMIN Clinical Trials Registry (UMIN-CTR; ID = UMIN000014081). Registered 27 May 2014.

## Background

On March 11, 2011, a huge earthquake hit northeast Japan, and the Pacific coast of this region suffered from a tsunami, with 15,882 casualties and 2,668 still missing. The tsunami also hit heavily the Tokyo Electric & Power Company Fukushima Dai-ichi nuclear plant. The plant lost control over regulating its nuclear reactors, which resulted in several explosions of the reactors and a discharge of radioactive substance, which flowed widely to a west-to-northwest direction from the plant. World Health Organization (WHO) and United Nations (UN) reports have estimated a cumulative effective dose in the first year following the accident for adults at 1–10 micro Sv in the sea-side area and other less affected areas of Fukushima prefecture [1, 2].

An important health problem after past nuclear power plant accidents was mental health among community residents in the affected area [3]. In past nuclear power plant accidents, a prolonged increase of non-clinical psychological distress such as depression and anxiety was observed among evacuee residents for many years after the accident [3, 4]. In the Chernobyl Nuclear Plant Accidents, middle-aged adults living in contaminated villages had greater psychological distress, sleep problems, and fatigue, than non-exposed controls at 4 years after the Accidents [5]. A similar pattern was observed in a follow-up survey at 6.5 years after the accident [6, 7]. Young mothers with small children are particularly known as a high risk group. In the Three Mile Island (TMI) nuclear plant accident, mothers of preschool children living within 10 miles of the nuclear power plant had an excess risk of experiencing clinical episodes of anxiety and depression during the year after the accident [8]. The same group was reported to have greater levels of subclinical symptoms of anxiety and depression at 1–2 years [9] and 10 years [10] after the accident. Evacuee mothers from exposed areas were reported to have higher symptom scores and poorer self-rated health at 11 years after the accident, compared to the controls [11], and they also were reported to have greater psychological distress [12, 13]. For the Fukushima Dai-ichi Nuclear Power Plant accident, it was also reported that mothers with infant and preschool children living in areas close to the plant experienced increased levels of depression [14]. Thus mothers with small children living in the Fukushima prefecture after the Nuclear Power Plant accident may be an important target of a psychosocial intervention for improving and promoting health. Fear and worry of possible radiation exposure following a nuclear power plant accident may be natural and rational in most cases; however, some people could have excessive fear or worry and be at risk for developing chronic depression and anxiety, which may interfere their daily life. However, no psychological intervention program has been developed or tested for effectiveness in reducing psychological distress among mothers with small children due to radiation anxiety after a nuclear power plant accident.

A psychological mechanism underlying excessive worry of exposure to radiation and related psychological distress of community residents has not been well-established yet. One potentially effective psychological intervention for improving radiation anxiety and its effect on mental health in a non-clinical population could be a treatment program of behavioral activation (BA). BA is a therapeutic process to increase pleasurable and rewarding activities using behavioral strategies such as activity scheduling [15]. Behavioral activation may be superior to the cognitive behavioral (CB) model, which has been widely applied to health anxiety [16]. In a five-part CB model for radiation anxiety, external cues such as media information or rumor of a health consequence of radiation (situation) are supposed to trigger people’s thoughts and beliefs of perceived risk of exposure to radiation. Such thoughts or beliefs could cause emotional reactions, such as anxiety and depression, increase physical symptoms through increased awareness, and influence people’s behaviors such as decreasing daily activities. These emotional, physical, and behavioral reactions could interplay and also increase negative thoughts or beliefs on radiation exposure. The common CB approach of changing dysfunctional belief on health anxiety (i.e., cognitive restructuring) [17] may not be effective in reducing radiation anxiety among mothers in Fukushima, because the beliefs of possible radiation exposure can be realistic and impossible to deny given the health risk of radiation exposure, as supported by the latest research evidence.

Previous intervention studies have shown a significant positive treatment effect of BA on depression in a clinical setting. A meta-analysis of randomized controlled trials (RCT) reported that BA improved depressive symptoms of depressed patients and community residents with depressive symptoms (Cohen’s *d* = 0.87, 95 % confidence interval [CI] = 0.60 to 1.15), as compared with control conditions [18]. Another meta-analysis of BA treatment for depression among clinical populations reported that the standardized mean difference (SMD) was 0.74 (95 % CI = 0.56 to 0.91) [19]. BA, often combined with other psychological interventions, shows significant effect on improving subthreshold depressive symptoms in the previous meta-analysis among non-clinical population [20]. There was no previous research testing the effectiveness of BA for health anxiety. However, in the CB model of radiation anxiety as described above, increasing pleasant daily activities is expected to decrease negative cognition and selective awareness/vigilance to adverse health effects of radiation exposure, improve psychological distress and physical symptoms, and stop the vicious cycle, without challenging thoughts and beliefs on radiation [21]. Behavioral activation seems a promising intervention to improve mental health among community residents with radiation anxiety after a nuclear power plant accident. However, there was no such intervention study after a nuclear power plant accident.

The purpose of this study was to examine the effectiveness of a BA-based program for improving psychological distress and physical symptoms among mothers with preschool children in Fukushima city and neighboring municipalities who were supposed to be with high levels of radiation anxiety and these symptoms 3 years after the Fukushima Daiichi Nuclear Power Plant accident, with a randomized controlled trial design.

## Methods

### Trial design

The study was a randomized controlled trial. The allocation ratio of the intervention group to the control group was 1 to 1. The study protocol was registered at the UMIN Clinical Trials Registry (UMIN-CTR; ID = UMIN000014081). The Research Ethics Review Board of Graduate School of Medicine/Faculty of Medicine, the University of Tokyo approved the study procedures (no. 10260). This manuscript was reported according to the CONSORT guideline checklist.

### Participants

The target population was mothers living with a preschool child(ren) in Fukushima-city or neighboring smaller municipalities (*n* = about 12,000), where radiation levels were similar (1–10 micro Sv in April 2011) and residents were supposed to have a similar level of radiation anxiety. All participants were recruited through an advertisement at a local newspaper, and posters and flyers posted at the public health center, all kindergartens and a large workplace in Fukushima city, and asked to contact a clinical research coordinator via phone or e-mail. Inclusion criteria were (a) mothers who have one or more children of preschool age, (b) living in Fukushima city or a neighboring municipality. Exclusion criteria were (a) having pre-existing health problems, or (b) being at risk for new or exacerbated health problems as a result of participating in the BA based intervention program of the present study. Study purposes and procedures were explained and written informed consent was obtained from the participants prior to the initiation of the study. After the 3-month follow-up survey, participants received a coupon worth 1,000 JPY as a reward for participation. No information was available concerning the recruitment route.

### Intervention

Participants who were allocated to the intervention group received the newly developed group-based behavioral activation program called *the liveliness enhancement workshop for mothers*. The program was a two-part workshop to provide a behavioral activation technique on enhancing participants’ liveliness, using an original 16-page textbook including an exercise worksheet for homework. This program was structured into two 90-min lessons, with one lesson given per week. In each lesson, participants share their own situation and discuss it with all participants including the workshop facilitator (clinical psychologist). At the end of each lesson, participants were asked to complete homework to facilitate their understanding until the next lesson. In the next lesson, participants received feedback to their homework from the workshop facilitator.

One of the unique features of the program was that the program was customized for mothers in two ways. The first was that we used examples in the textbook that the mothers often experience in their daily lives. The second was that the program included the work that teaches how they can get over difficulties related to child rearing (i.e., not enough time to carry out a plan of the behavioral activation). This program included case-formulation skills based on the cognitive behavioral model (in Lesson 1) and behavioral activation skills (in Lessons 1 and 2). In Lesson 1, participants first learn about the cognitive behavioral (CB) model, especially the five-part model referring to five areas: situation, thoughts, emotions, behavior, and physical feelings) [21] and a self-case formulation based on this model. After that, participants learn about a theory of behavioral activation and how to plan an activity schedule for enhancing liveliness. In Lesson 2, participants share and discuss results of their homework and any barriers they experienced in completing homework. After that, participants plan their own self-care strategy.

### Intervention group

Participants in the intervention group received two weekly workshops at a local health center in Fukushima city. During the Lessons 1 and 2, participants were asked to do their own homework. Participants could leave their children at a day-care center in the same building. For a process evaluation, levels of understanding the content and satisfaction with the intervention program ware assessed at the end of the Lesson 2.

### Control group

Participants in the control group were treated as waiting list and received no specific intervention activities. Any active treatment was not provided to the participants in the control group during the 3-month follow-up period, because they were non-clinical status. Participants in the control group were provided with a same workshop after the 3-month follow-up.

### Outcome measurements

All outcomes were measured using a self-report questionnaire at baseline, 1-month, and 3-month follow-up, which are time frames often used in previous intervention studies of BA [19].

### Primary outcomes

#### Psychological distress

A self-report scale, K6 [22, 23], was used to measure psychological distress. The K6 scale consists of six items assessing the frequency (0 [*none of the time*] to 4 [*all of the time*]) with which respondents reported the degree of psychological distress during the past 30 days. The total score ranges from 0 to 24. The internal reliability and validity found in previous studies are acceptable [22].

#### Physical symptoms

Physical symptoms were measured by the Brief Job Stress Questionnaire (BJSQ) [24], comprising 10 items assessing headaches, heart palpitations, dizziness, among others, with a four-point response scale, from 1 (*never*) to 4 (*almost always*). The total scores range from 10 to 40.

### Secondary outcomes

#### Radiation anxiety

A scale was newly developed to capture radiation anxiety, which is defined as fears and worries of health-related and other problems due to possible radiation exposure, particularly targeting community residents in Fukushima [25]. This scale consists of seven items asking about the respondents’ fears and worries of effects of radiation exposure on their own health and the next generation’s health, and the effect of news reports on the accident at the nuclear power plant, among others. The response options (item scores) were on a four-point scale, from *totally disagree* (1) to *totally agree* (4). The total scale scores ranged from 7 to 28. Reliability and validity of the scale was tested in a sample of 141 randomly sampled residents of Fukushima city in an earlier report. Cronbach’s alpha coefficient for the total score was 0.81.

#### Liveliness

Liveliness was measured by the subscale of the multiple mood scale, comprising 10 items [26]. The response options ranged from 1 (*not at all*) to 4 (*clearly*).

#### Life satisfaction

Participants were asked to rate their satisfaction of life with a visual analogue scale (VAS) ranging from 0 (*completely dissatisfied*) to 100 (*completely satisfied*). Life satisfaction was assessed by asking participants, “How would you rate your satisfaction of your current life?”

### Sample size

A required sample size was calculated for one of the outcome variables, i.e., psychological distress. A meta-analysis of behavioral activation treatment for depression yielded a standardized mean difference (SMD) of -0.74 (95 % CI: -0.91 to -0.56) at post-test [19]. To detect an effect size of 0.74 or more at an alpha error rate of 0.05 and a beta error rate of 0.25, the estimated sample size was 24 participants in each group. With anticipating the dropout rate of 20 %, the necessary sample size was 30 participants per arm. The statistical power was calculated using the G*Power 3 program [27, 28].

### Randomization

Participants who fulfilled the inclusion criteria were randomly allocated to intervention or control groups. Permuted-block randomization was conducted. A permuted-block random table was generated by NK. A clinical research coordinator conducted enrollment. An independent research assistant conducted the assignment. The permuted-block random table was password-protected and blinded to the researcher. Only the research assistant had access to it during the work of random allocation.

### Statistical analyses

An interaction effect of a group (intervention and control) × time (baseline and 1-month or 3-month follow-up) was estimated using a mixed model for repeated measures analysis of variance model analysis (ANOVA), as an indicator of intervention effect at each follow-up. The analysis was conducted by intention-to-treat. We used SPSS Statistics 21.0 (IBM Corp., USA). Also, we calculated Cohen’s *d* and the 95 % confidence intervals only among those who completed the questionnaire at baseline and at follow-up, although the effect sizes may be more biased because of dropping out. We interpreted the effect size following suggested criteria, i.e, small (0.2), medium (0.5), and large (0.8) [29].

## Results

### Recruitment

**Fig. 1:**
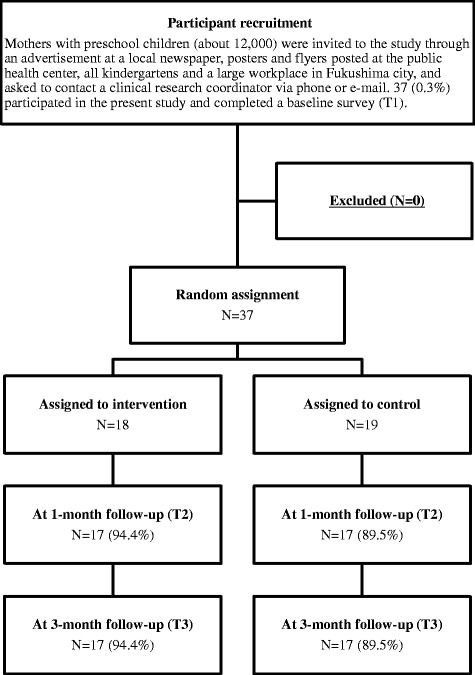
Participant flowchart

Recruitment and the baseline survey were conducted in August 2014. Both groups were surveyed at 1-month (September 2014) and 3-month follow-up (November 2014). A participant flowchart is shown in Fig. 1. Thirty-seven mothers participated in the study and completed a baseline survey. Nobody had to be excluded. One participant was from a neighboring municipality; all others were living in Fukushima-city. All 37 participants were randomly assigned to either an intervention or a control group (18 in the intervention group and 19 in the control group, respectively). At 1-month follow-up, 17 (94.4 %) participants in the intervention group and 17 (89.5 %) in the control group completed the follow-up survey. At the 3-month follow-up, 17 (94.4 %) participants in the intervention group and 17 (89.5 %) in the control group completed the follow-up survey. Reasons for dropping out were poor physical condition of a family member, parturition, or moving.

### Baseline characteristics

Demographic characteristics are presented in Table 1. Participants who were assigned to the control group tended to be older and have lower levels of education than those of the intervention group. In the whole sample, more than half of the participants had two children.

**Table 1:** Baseline characteristics of participants in the intervention and control groups

	Intervention group (*N* = 18)		Control group (*N* = 19)	
	n (%)	Average (SD)	n (%)	Average (SD)
Age (years)		33.5 (3.5)		37.5 (4.1)
Marital status				
Married	18 (100.0)		17 (89.5)	
Missing	0 (0.0)		2 (10.5)	
Occupational status				
Regular employment	1 (5.6)		2 (10.5)	
Non-regular employment	1 (5.6)		1 (5.3)	
On maternal leave	4 (22.2)		2 (10.5)	
Housewife	12 (66.7)		12 (63.2)	
Missing	0 (0.0)		2 (10.5)	
Education				
High school	0 (0.0)		3 (15.8)	
Some college	8 (44.4)		10 (52.6)	
University	9 (50.0)		4 (21.1)	
Graduate school	1 (5.6)		1 (5.3)	
Missing	0 (0.0)		1 (5.3)	
Number of children		1.7 (0.7)		1.9 (0.8)^a^

^a^The number of respondents was 17

### Effects of the intervention on outcome variables

Table 2 shows the means and standard deviations of the outcome variables at baseline, 1-month, and 3-month follow-ups in the intervention and control groups. Table 3 shows the estimated effects of the behavioral activation intervention program on the outcome variables on the basis of the mixed-model analysis. The present BA program failed to show a significant intervention effect on primary outcomes. Only marginally significant effects were shown on psychological distress (*t* = -1.99, *p* = 0.051) and physical symptoms (*t* = -1.83, *p* = 0.07) at 1-month follow-up. The effect sizes were medium to large and only K6 was significant (-0.72 [95 % CI -1.36 to -0.09] on the K6 and -0.56 [95 % CI -1.21 to 0.08] on the BJSQ, respectively).

**Table 2:** Means (SDs) of outcome variables at baseline, 1- and 3-month follow-up in the intervention and control groups for the whole sample

	Intervention			Control		
	Baseline (*N* = 18)	1-month (*N* = 17)	3-month (*N* = 17)	Baseline (*N* = 19)	1-month (*N* = 17)	3-month (*N* = 17)
	mean (SD)	mean (SD)	mean (SD)	mean (SD)	mean (SD)	mean (SD)
Psychological distress	5.8 (3.8)	4.0 (2.4)	5.6 (4.7)	10.0 (3.9)	10.6 (5.6)	10.4 (5.6)
Physical symptoms	17.1 (4.7)	16.1 (4.5)	17.2 (4.6)	20.1 (4.1)	21.5 (5.8)	20.2 (5.8)^b^
Radiation anxiety	16.4 (4.8)	14.5 (4.0)	15.2 (4.4)	18.7 (5.1)	18.4 (5.6)	18.2 (5.2)
Liveliness	24.8 (6.7)^a^	28.0 (5.5)	27.5 (6.0)	22.7 (4.8)	23.8 (7.2)	20.8 (5.4)
Life satisfaction	58.5 (25.2)	68.5 (21.3)	68.7 (25.5)	39.2 (15.9)	39.5 (21.6)	39.3 (21.3)

^a^The number of respondents was 17

^b^The number of respondents was 16

**Table 3:** Effect of the behavioral activation program on outcome variables for the whole sample

	Follow-up	Estimates of fixed effects (95 % CI)	t	*P*	d (95 % CI)
Psychological distress	1 month	-2.85 (-5.71 to 0.01)	-1.99	0.051	-0.72 (-1.36 to -0.09)
	3 month	-0.90 (-4.05 to 2.25)	-0.58	0.57	-0.28 (-0.96 to 0.39)
Physical symptoms	1 month	-2.37 (-4.96 to 0.22)	-1.83	0.07	-0.56 (-1.21 to 0.08)
	3 month	-0.15 (-3.40 to 3.10)	-0.09	0.93	0.03 (-0.68 to 0.73)
Radiation anxiety	1 month	-0.93 (-2.23 to 0.36)	-1.44	0.16	-0.41 (-1.08 to 0.26)
	3 month	-0.15 (-1.92 to 1.62)	-0.17	0.87	-0.02 (-0.72 to 0.68)
Liveliness	1 month	2.17 (-1.39 to 5.73)	1.22	0.23	0.40 (-0.31 to 1.10)
	3 month	4.59 (0.88 to 8.29)	2.52	0.02	0.88 (0.25 to 1.52)
Life satisfaction	1 month	9.01 (-1.33 to 19.36)	1.75	0.09	0.67 (0.03 to 1.31)
	3 month	9.40 (-1.42 to 20.22)	1.76	0.09	0.56 (-0.09 to 1.22)

On the secondary outcomes, the BA program showed a significant intervention effect on increasing liveliness at 3-month follow-up, and a marginally significant effect on life satisfaction at 1-month and 3-month follow-up. On the other hand, the BA program did not show a significant intervention effect on improving radiation anxiety.

There were a few subgroup differences observed in the effect of the BA program: the effect sizes were greater for employed mothers (*n* = 6 in the intervention group and *n* = 5 in the control group) for psychological distress (-1.15 [95 % CI -2.22 to -0.08]), liveliness (1.47 [95 % CI 0.66 to 2.27]) and life satisfaction (1.19 [95 % CI 0.21 to 2.17]) at 3-month follow-up. The effect size was greater among housewives (*n* = 12 in each group) for life satisfaction (1.05 [95 % CI 0.32 to 1.77]) at 1-month follow-up. The effect sizes were greater among mothers who had university graduates or higher education (*n* = 10 in the intervention group and *n* = 5 in the control group) for liveliness (1.14 [95 % CI 0.21 to 2.08]) at 3-month follow-up and life satisfaction (1.56 [95 % CI 0.83 to 2.28]) at 1-month follow-up. The effect sizes were greater among mothers with two or more children for psychological distress (*n* = 10 in the intervention group and *n* = 12 in the control group) at 1-month (-1.24 [95 % CI -1.94 to -0.55]) and 3-month (-0.93 [95 % CI -1.73 to -0.14]), and for liveliness (1.12 [95 % CI 0.34 to 1.89]) at 3-month follow-up.

### Process evaluation

All of the participants (*N* = 18) in the intervention group received the two lessons of the group-based BA program. A total of 17 (94.4 %) responded that the behavioral activation program was *easy* to *moderately easy* to understand; 17 (94.4 %) reported the program useful; and 18 (100 %) rated their satisfaction with the program as *very much* or *mostly*.

## Discussion

The present RCT examined the effects of a newly developed BA-based program on improving psychological distress and physical symptoms at 1- and 3-month follow-ups among mothers with preschool children in Fukushima city and neighboring municipalities. Marginally statistically significant intervention effects were observed for the two primary outcomes (e.g., psychological distress and physical symptoms) at 1-month follow-up, with medium to large effect sizes. The BA program showed a significant intervention effect on improving a secondary outcome, liveliness, at 3-month follow-up with a large effect size. This BA program may be effective on improving psychological distress, physical symptoms and psychological well-being among mothers with preschool children after the nuclear power plant accident in Fukushima, even though a further large-scale study is required to confirm the intervention effects.

To our knowledge, the present study is the first to examine whether a BA-based program could improve psychological distress and physical symptoms that were affected by radiation anxiety in a non-clinical community population. However, there were only marginally significant effects on psychological distress and physical symptoms at 1-month follow-up. A possible explanation for the lack of effect of the present BA-based intervention could be the low intensity and short duration of the intervention. A previous meta-analysis reported that the median number of clinical sessions with a therapist was eight and the number of sessions was not associated with effect size [19]. However, the mean number of sessions was 8.5 (SD = 3.6) and in 24 of 26 studies the mean number of sessions was five or more. Compared with those results, the BA intervention of the present study could be seen as too limited in duration and intensity to improve psychological and physical outcomes. On the other hand, the effect sizes on these outcomes could be acceptable, and a significant effect size was observed on improving psychological distress of those participants who completed the questionnaire at baseline and at follow-up. In consultation with the target population of the present study, five sessions or more were considered not to be feasible. Moreover, using a high-intensity intervention program may decrease participation or the completion rate of participants.

The present study failed to show significant effects of the BA program on improving radiation anxiety at 1- or 3-month follow-ups. However, radiation anxiety tended to improve at 1-month follow-up to some extent, as also did physical symptoms and psychological distress. A possible process of health anxiety causing hypochondriasis is that excessive anxiety about one’s health leads to believing oneself to be sick, because of misinterpretation of one’s physical symptoms as a signal of a serious health problem [17]. The result could lead to further anxiety, increased autonomic symptoms, and urges to seek medical evaluation for a suspected health problem. This process often prevents patients from correcting their overestimation of health-related threats, thus leading to the persistence of hypochondriasis. According to this process [17], improvement of physical symptoms or psychological distress would lead to cognitive changes about health-related threats, as in the perception of radiation anxiety in this case. The present finding supports a view that the theory of health anxiety [17] is also applicable to post-disaster radiation anxiety. Another explanation is also possible, namely, that pleasant activities increased by BA altered the participants’ perceptions of risks connected with radiation, thus decreasing physical or psychological symptoms resulting from anxiety. It may be promising to test whether a BA-based intervention is useful for improving excessive radiation anxiety after a nuclear power plant accident in future research.

In the present study, liveliness significantly improved at 3-month follow-up; and life satisfaction marginally significantly improved at 1- and 3-month follow-ups. A previous meta-analysis reported that BA interventions significantly improved well-being [30]. The present BA program was designed particularly to encourage participants to increase pleasant activities in their daily life, rather than activities improving their negative mood. This may be a reason that the effects were more prominent on these well-being outcomes than on physical symptoms and psychological distress. The BA program may be effective for improving psychological well-being of mothers with preschool children after a nuclear power plant accident.

Based on the subgroup analyses, participating mothers who were employed, highly educated, or had two or more children seemed to enjoy benefits from the present BA program for a longer term (i.e., three months). The BA program may remain more effective for employed mothers, since they may have more chance to exercise BA in a wider social network including their workplaces. The BA program may be more effective for mothers with higher education, because of their better understanding the program contents. The BA program may be more effective for mothers with multiple children. They may have more worry and burden in rearing their children under radiation anxiety, and thus feel a greater need to learn the BA program. Unfortunately, due to the small sample size, the present study could not confirm these subgroup differences, that should be fully investigated in future research.

Possible limitations of the present study should be considered. A major limitation of this study was the small sample size. The sample size of the present study was too small. Preliminary sample size calculation showed that the necessary sample size was 30 participants per arm, while the present study had hardly two-thirds that number. The present study may have overlooked some effects of the BA program because of the limited statistical power. Second, participants were recruited from Fukushima city and neighboring municipalities, not from the entire aria of Fukushima prefecture. Most participants were housewives, and had a higher education. Participants in the present study may not be representative of mothers with radiation anxiety in the whole Fukushima prefecture. Therefore, the generalizability of the present findings may be somewhat limited. Third, all outcomes in the present study were measured by self-report, which may be affected by the perception of participants or by situational factors. A self-reported measure could be vulnerable to a cognitive bias. If participants answered the questionnaire in a socially desirable manner, the observed association may have been overestimated. Fourth, residence year of participants were not assessed. Fifth, follow-up period in this study was limited to estimate its long-term effect. Sixth, the present study did not examine the cost-effectiveness of the BA program. Seventh, there may be heterogeneity of the effect of the present BA program depending on educational attainment, employment status, and the number of children of participating mothers, which we could not investigated in detail in this study because of the small sample size. A further RCT should be conducted to examine whether the BA program is effective in a larger representative sample of mothers living in the whole Fukushima prefecture with a long-term follow-up.

## Conclusions

This BA program may be effective for improving psychological distress, physical symptoms, and well-being, at least for a short duration, among mothers with preschool children after the nuclear power plant accident in Fukushima, while a further large-scale study is needed. This program may possibly contribute to improve health and well-being of mothers living in Fukushima, provided by trained public health nurses at each local public health center in Fukushima prefecture.

## Abbreviations

BA: Behavioral activation
BJSQ: Brief job stress questionnaire
CB: Cognitive behavioral
K6: Kessler’s Psychological Distress Scale
RCT: Randomized controlled trial
SMD: Standardized mean difference
TMI: Three Mile Island
UN: United Nations
VAS: Visual analogue scale
WHO: World Health Organization
